# Genomic diversity and metabolic potential of marine *Pseudomonadaceae*

**DOI:** 10.3389/fmicb.2023.1071039

**Published:** 2023-04-06

**Authors:** Léa Girard, Cédric Lood, René De Mot, Vera van Noort, Julia Baudart

**Affiliations:** ^1^Centre of Microbial and Plant Genetics, KU Leuven, Leuven, Belgium; ^2^Department of Biosystems, Laboratory of Gene Technology, KU Leuven, Leuven, Belgium; ^3^Institute of Biology, Leiden University, Leiden, Netherlands; ^4^Laboratoire de Biodiversité et Biotechnologie Microbiennes, Sorbonne Université, CNRS, Observatoire Océanologique, Banyuls-sur-Mer, France

**Keywords:** *Pseudomonas chaetocerotis* sp. nov., *Pseudomonas*, *Halopseudomonas*, *Stutzerimonas*, genome mining of natural products, phylogenetics

## Abstract

Recent changes in the taxonomy of the *Pseudomonadaceae* family have led to the delineation of three new genera (*Atopomonas*, *Halopseudomonas* and *Stutzerimonas*). However, the genus *Pseudomonas* remains the most densely populated and displays a broad genetic diversity. *Pseudomonas* are able to produce a wide variety of secondary metabolites which drives important ecological functions and have a great impact in sustaining their lifestyles. While soilborne *Pseudomonas* are constantly examined, we currently lack studies aiming to explore the genetic diversity and metabolic potential of marine *Pseudomonas* spp. In this study, 23 *Pseudomonas* strains were co-isolated with *Vibrio* strains from three marine microalgal cultures and *rpoD*-based phylogeny allowed their assignment to the *Pseudomonas oleovorans* group (*Pseudomonas chengduensis*, *Pseudomonas toyotomiensis* and one new species). We combined whole genome sequencing on three selected strains with an inventory of marine *Pseudomonas* genomes to assess their phylogenetic assignations and explore their metabolic potential. Our results revealed that most strains are incorrectly assigned at the species level and half of them do not belong to the genus *Pseudomonas* but instead to the genera *Halopseudomonas* or *Stutzerimonas*. We highlight the presence of 26 new species (*Halopseudomonas* (*n* = 5), *Stutzerimonas* (*n* = 7) and *Pseudomonas* (*n* = 14)) and describe one new species, *Pseudomonas chaetocerotis* sp. nov. (type strain 536^T^ = LMG 31766^T^ = DSM 111343^T^). We used genome mining to identify numerous BGCs coding for the production of diverse known metabolites (i.e., osmoprotectants, photoprotectants, quorum sensing molecules, siderophores, cyclic lipopeptides) but also unknown metabolites (e.g., ARE, hybrid ARE-DAR, siderophores, orphan NRPS gene clusters) awaiting chemical characterization. Finally, this study underlines that marine environments host a huge diversity of *Pseudomonadaceae* that can drive the discovery of new secondary metabolites.

## Introduction

*Pseudomonas* spp. are ubiquitous bacteria able to colonize a wide range of environments such as soil, stream and ground waters or associated to plants (Ramos, 2004). Their occurrence in soil or associated to diverse plant crops is constantly examined and their implication in fundamental ecological processes is well established (Wasi et al., 2013; Dignam et al., 2019; Yadav et al., 2021). So far, *Pseudomonas* spp. have been randomly isolated from seawater, sediments or associated to higher organisms, demonstrating that they naturally occur in marine environments and occasionally represent a large majority of the studied bacterial communities (Amer et al., 2015; Jiang et al., 2015; Viggor et al., 2015; Berner et al., 2018). Several *Pseudomonas* species are also fish pathogens and have detrimental impacts on aquaculture around the world (e.g., *P. plecoglossicida*, *P. baetica*, *P. stutzeri*, *P. anguilliseptica* or *P. alcaligenes*) (Lopez et al., 2012; Xu et al., 2015; Wiklund, 2016; Beaton et al., 2018). However, most of the studies are using 16S rRNA gene sequences to perform taxonomic affiliations and this gene is not sufficiently discriminant to differentiate *Pseudomonas* strains at the species level, which results in misidentifications and underestimations of diversity (Amer et al., 2015; Jiang et al., 2015; Viggor et al., 2015; Berner et al., 2018).

The genus *Pseudomonas*, originally made of several groups and subgroups (Lalucat et al., 2020; Girard et al., 2021), was recently divided into four and the *Pseudomonadaceae* family now contains three supplementary genera (*Atopomonas*, *Halopseudomonas* and *Stutzerimonas*) (Rudra and Gupta, 2021; Lalucat et al., 2022). The genera *Halopseudomonas* and *Stutzerimonas* are corresponding to the former *P. pertucinogena* and *P. stutzeri* groups, while the genus *Aptomonas* only includes one species, *A. hussainii* (previously *P. hussaini*). Consequently, the genus *Pseudomonas* is now made of 14 groups (*P. aeruginosa*, *P. anguilliseptica*, *P. fluorescens*, *P. linyingensis*, *P. lutea*, *P. massiliensis*, *P. oleovorans*, *P. oryzihabitans*, *P. pohangensis*, *P. putida*, *P. resinovorans*, *P. rhizosphaerae*, *P. straminea* and *P. syringae*) and several orphan groups (Girard et al., 2021).

In order to thrive in an extremely wide range of environments *Pseudomonas* spp. display a great metabolic diversity and their metabolite production appears to be an important strategy in sustaining their lifestyles (Kraemer, 2004; Götze and Stallforth, 2019; Girard et al., 2020a). Indeed, these molecules are often involved in important ecological functions (e.g., iron-scavenging, swarming motility, biofilm formation, pathogenicity, cooperation or antagonism) and a large majority have anti-microbial properties (anti-bacterial, anti-fungal or anti-viral) (Gross and Loper, 2009). *Pseudomonas* spp. have the ability to assemble intriguing compounds and their metabolic potential his supported by the prevalence, in their genomes, of very diverse biosynthetic systems [e.g., non-ribosomal peptide synthetases (NRPSs), polyketides synthases (PKSs, hybrid systems) but also by the presence of many orphan Biosynthetic Gene Clusters (BGCs)] encoding for the production of unknown metabolites (Gross and Loper, 2009; Girard et al., 2020a). Over the last decade, the expansion of affordable sequencing technologies and advances in bioinformatics has led to the genome mining (or genome-guided) discovery of a wide diversity of compounds (de Bruijn et al., 2007) and marine bacteria were shown to produce novel secondary metabolites with unique chemical structure leading the development of new drugs (Petersen et al., 2020). Marine *Pseudomonas* were recently pinpointed as a prolific source for molecules of biotechnological interest (Carroll et al., 2019; Bollinger et al., 2020), however, studies linking accurate taxonomic affiliation and metabolic potential of strains are still missing.

In this study, we used the *rpoD* gene to taxonomically assign twenty-three *Pseudomonas* strains co-isolated with *Vibrio* strains from three marine microalgal cultures: *Chaetoceros calcitrans*, *Chaetoceros gracilis* and *Isochrysis galbana affinis* Tahiti. We performed whole genome sequencing on three selected strains and inventoried all marine *Pseudomonas* isolates with available genomic sequences. We then used whole genome analyses (Average Nucleotide Identity (ANIb), digital DNA–DNA hybridization (dDDH)) to taxonomically re-assign 77 marine strains previously identified as *Pseudomonas* sp., *P. fluorescens* or *P. putida*. Based on a polyphasic approach, we show that two isolates, 536 and 293, represent a novel species within the *P. oleovorans* group, *Pseudomonas chaetocerotis* sp. nov. (type strain 536^T^ = LMG 31766^T^ = DSM 111343^T^). Ultimately, we used a combination of online tools and phylogenetic analyses to explore the metabolic potential of marine *Pseudomonas* and give an overview of the nature and diversity of their BGCs.

## Materials and methods

### Isolation and culture conditions

The diversity of *Vibrio* species associated to three marine microalgal species, *Chaetoceros calcitrans*, *Chaetoceros gracilis* and *Isochrysis galbana affinis* Tahiti, was examined between February and June 2018. Microalgal cultures were directly plated on Thiosulfate-Citrate-Bile salts-Sucrose (TCBS) and incubated at 25°C for 48 h. *Vibrio* typically grow on TCBS as large green or yellow colonies. *Pseudomonas* strains appear as small green colonies on TCBS and as small cream colonies on marine agar (MA). A total of 125 colonies were picked randomly and isolated on MA plates. After isolation, strains were identified based on the *gyrB* gene, suitable for taxonomic identification of *Vibrio* isolates [data not shown; (Girard et al., 2018)], and revealed the presence of 23 *Pseudomonas* isolates. Therefore, the taxonomic affiliations of *Pseudomonas* isolates were assessed using the *rpoD* gene (Girard et al., 2020b).

### *rpoD* sequencing

PCR reactions were performed as previously described in Girard et al. (2020a). The cell lysates of the 23 *Pseudomonas* strains were used as PCR templates. PCR amplifications were performed using the primers PsEG30F and PsEG790R (Mulet et al., 2009), and KAPA2G Fast HotStart ReadyMix (Sigma–Aldrich, Saint-Louis, Missouri, United States). Cycling conditions were as follows: initial denaturation at 95°C for 5 min followed by 30 cycles of annealing at 60°C for 30 s, extension at 72°C for 30 s and denaturation at 95°C for 15 s, and reactions were completed at 72°C for 2 min. PCR products were purified using the GenElute PCR Clean-Up kit (Sigma–Aldrich Saint-Louis, Missouri, United States). Purified PCR products were then sequenced using the same set of primers (PsEG30F and PsEG790R) by Sanger sequencing (Macrogen Europe, Amsterdam, The Netherlands) to obtain a final fragment of approximately 650 bp.

### Phenotypic, biochemical and chemotaxonomic characterization

*Pseudomonas* strain 536^T^ was grown on LB agar for 24 h at 30°C and cell morphology and flagellation were observed by using a HITACHI type H7500 transmission electron microscope and a negative-staining technique. For negative staining, *Pseudomonas* strain 536^T^ was fixed with 2.5% glutaraldehyde and stained with 0.1% uranyl acetate. Growth in LB broth for 2 days was assessed at 4, 16, 25, 28, 37 and 41°C, at pH 5, 6, 8 and 10 and with 0, 1, 2, 4, 5, 8 and 10% (w/v) NaCl. Phenotypic characterization was assed using the GEN III MicroPlate (Biolog) following the manufacturer’s instruction and the API 20 NE kit (bioMérieux) in LB at 30°C. Whole-cell fatty acids composition was determined by FAME (Fatty Acid Methyl Ester) and respiratory quinones were extracted, and confirmed using HPLC (Collins and Jones, 1981; Sasser, 2001). *P. toyotomiensis* JCM 15604^T^ and *P. chengduensis* DSM 26382^T^ were used as reference strains and assessed in the same growth conditions.

### Whole genome sequencing

In order to validate the *rpoD*-based taxonomic affiliation, the genome of one representative strain for each *rpoD* cluster, namely 402, 536 and 718, was sequenced. Genomic DNA was extracted using the Gentra Puregene Yeast/Bact. Kit (Qiagen, Hilden, Germany). Nextera XT library preparation kit and the Illumina MiSeq sequencer were used for genome sequencing (BASECLEAR, Leiden, The Netherlands). Libraries were sequenced using a paired-end approach (2 × 150 bp) and the genome coverage was routinely above 40 X. The quality of the Illumina reads was assessed using FastQC v. 0.11.9 and Trimmomatic v. 0.38 for adapter clipping, quality trimming (LEADING:3 TRAILING:3 SLIDINGWINDOW:4.15), and minimum length exclusion (>50 bp) (Bolger et al., 2014). *De novo* genome assembly was performed with the SPAdes assembler v. 3.13.0 (Bankevich et al., 2012). Strain 536^T^ was re-sequenced using Nanopore technology (Oxford Nanopore Technology, London, United Kingdom). The quality of the dataset of nanopore reads was assessed with the NanoPack software suite (De Coster et al., 2018), and combined with Illumina using the hybrid assembler Unicycler v0.4.8 (Wick et al., 2017).

### Phylogenetic and whole genome analyses

We inventoried all genomic sequences of *Pseudomonas* strains isolated from marine sources available on NCBI. We searched among three categories, unaffiliated *Pseudomonas* sp. strains (November 2021),^1^ and strains affiliated to *P. fluorescens* (November 2021)^2^ and *P. putida* (November 2021).^3^ Indeed, these two species also represent the largest groups within the *Pseudomonas* genus and NCBI affiliations at the species level are the most likely to be incorrect (Girard et al., 2021). The first phylogenetic analysis included the *rpoD* sequences of our 23 *Pseudomonas* isolates from marine microalgal cultures (Supplementary Table S1), the type strains of the *P. oleovorans* group and *P. anguilliseptica* (outgroup). The second *rpoD*-based phylogenetic analysis included 322 type strains (277 *Pseudomonas*, 23 *Halopseudomonas*, 14 *Stutzerimonas*, 7 other *Pseudomonadaceae* and *Cellvibrio japonicus* as the outgroup; Supplementary Table S2) and 61 marine *Pseudomonas* strains (Supplementary Table S3). The trees showing the phylogenetic relationships of environmental *Pseudomonas* isolates with the type strains were constructed using maximum-likelihood methods in MEGA-X (best evolutionary model; Tamura et al., 2011) and annotated with iTOL (Letunic and Bork, 2019). The genomes of type strains (275 *Pseudomonas*, 22 *Halopseudomonas*, 14 *Stutzerimonas*; Supplementary Table S2) and environemental strains (Supplementary Table S3) were used for whole genome analyses. Average nucleotide identity (ANI) values were calculated using the PYANI v0.2.10 with default parameters (Pritchard et al., 2016). When ANIb values were considered as ambiguous (i.e., between 95 and 96.5%), digital DNA–DNA Hybridization (dDDH) were calculated using the online tool, the Genome-to-Genome Distance Calculator GGDC (March 2022).^4^

### BGCs analyses

We first used antiSMASH 6.0 to identify and annotate secondary metabolites BGCs (Blin et al., 2021). The MIBiG cluster comparison and ClusterBlast packages, now allow to detect potential unexplored forms of BGCs but also to easily identify identical BGCs in other strains. As not every known BGCs (published) is yet registered in the database, it thus considerably facilitates identifications and comparisons. The synteny of each BGC was manually inspected. NRPS clusters were checked to verify the expected domain organization (i.e., siderophores, lipopeptides) and the online PKS/NRPS analysis tool^5^ was used to delineate and extract A-domains for amino-acid sequence predictions (Rokni-Zadeh et al., 2012; Ye et al., 2013; Girard et al., 2020a).

## Results and discussion

### *Pseudomonas* isolates from marine microalgal cultures

A total of twenty-three *Pseudomonas* strains were co-isolated with *Vibrio* species on TCBS agar plates from three marine microalgal cultures, *Chaetoceros calcitrans* (*n* = 9), *Chaetoceros gracilis* (*n* = 12) and *Isochrysis galbana affinis* Tahiti (*n* = 2), and identified using *rpoD* amplicon analysis (Supplementary Table S1). All isolates belong to the *Pseudomonas* genus, clustering within the *P. oleovorans* group and affiliated to two known species, *P. chengduensis* (*n* = 19) and *P. toyotomiensis* (*n* = 2), and one new *Pseudomonas* species (*n* = 2; Supplementary Figure S1). *Pseudomonas* strains were previously isolated from cyanobacterial or phytoplanktonic blooms (Ansari et al., 2015; Choi et al., 2016; Park et al., 2016; Berner et al., 2018; Sinha et al., 2019). However, this is the first study reporting *Pseudomonas* isolates from the *P. oleovorans* group associated with marine diatom species. Diatoms from the genus *Chaetoceros* are not only the most widespread and abundant in marine habitats worldwide but also the richest in term of number of species (Li et al., 2017) which suggests that *Pseudomonas* strains could evolve in *Chaetoceros* blooms in diverse marine environments. Furthermore, *Chaetoceros calcitrans*, *Chaetoceros gracilis* and *Isochrysis galbana affinis* Tahiti are the most frequently used species in aquaculture, especially as food in bivalve and shrimp marinicultures, but can also be used for the production of biodiesel (Brown et al., 1997; Kwangdinata et al., 2014; Marquez et al., 2019). Considering the economic and ecological importance of these microalgae and the tight relationships between *Pseudomonas* spp. and plants in terrestrial environments (commensal, pathogenic and biocontrol strains; Höfte and De Vos, 2007; Girard et al., 2020a), it would be of great interest to study in depth relationships between *Pseudomonas* spp. and marine diatoms. Finally, *Vibrio* spp. are the most studied bacteria, in terms of diversity, abundance and distribution, in marine environments around the world (Zhang et al., 2018). TCBS is a selective medium commonly use for the enumeration of cultivable *Vibrio* spp. from all kinds of samples. Previous studies have highlighted that other bacteria can grow on this medium, such as *Burkholderia cepacia*, *Aeromonas salmonicida*, *A. caviae*, *A. hydrophila*, *A. sobria*, *Chromobacterium violaceum*, *Listonella damsela*, *Shewanella putrefaciens*, *Flavobacterium meningosepticum* and *Pasteurella* sp. (Hervio-Heath et al., 2002). However, this is the first study reporting the isolation of different *Pseudomonas* species on TCBS.

### Taxonomic affiliations of marine *Pseudomonas*

**Figure 1:**
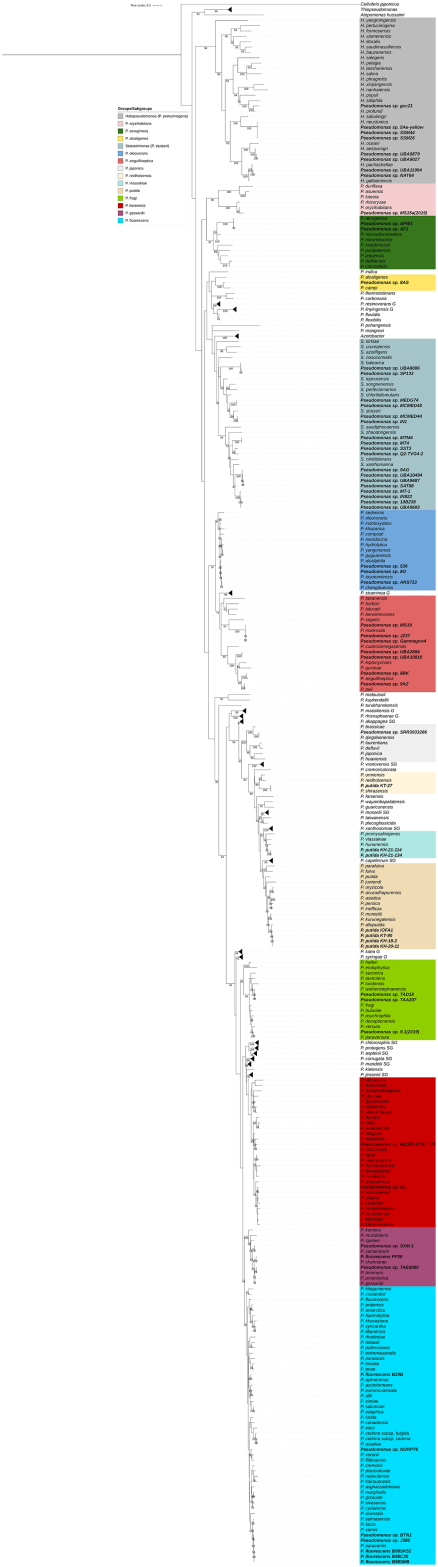
Phylogenetic tree of the *Pseudomonadaceae* based on the *rpoD* gene of type strains (Supplementary Table S2) and environment strains (Supplementary Table S3). The maximum likelihood phylogenetic tree was constructed using the GTR + G + I model (MEGA-X). Bootstrap values were calculated based on 1,000 replications and only bootstrap values higher than 50% are shown. Environmental strains are highlighted in bold. *C. japonicus* is used as the outgroup.

Three isolates were selected to confirm *rpoD*-based taxonomic affiliation and whole genome sequencing was performed on strains 536, 402 and 718. ANIb values confirmed that *Pseudomonas* strains 402 (96.60%) and 718 (97.37%) are belonging to, respectively, *P. chengduensis* and *P. toyotomiensis*, while strain 536 represents a new species and was included in the following dataset for further analyses. Most of NCBI *Pseudomonas* genomes were belonging to strains isolated from soils or plants and only 77 genomes of strains isolated from marine sources were available, 3.7% of *Pseudomonas* sp. (65/1775), 3.7% of *P. putida* (7/190) and 1.8% of *P. fluorescens* (5/272). A large majority of these genomes were obtained in the course of broad metagenomics projects (e.g., Tara Ocean) and thus are highly fragmented (Supplementary Table S3). Consequently, we were only able to retrieve the *rpoD* gene in 61 genomes and these 61 strains were thus included in the *rpoD*-based phylogeny (Figure 1). However, whole genome analysis allowed us to conclude on the taxonomic status of the 77 strains (e.g., ANIb, Table 1; Supplementary Table S4). Following the recent changes in the *Pseudomonadaceae* family, our analyses show that almost half of the strains were belonging to the genus *Pseudomonas* while the remaining are members of the newly described genera, *Halopseudomonas* (*n* = 12) and *Stutzerimonas* (*n* = 27) (Table 1; Figure 1). All strains previously classified as *P. putida* and *P. fluorescens* were incorrectly affiliated at the species level (Table 1). Solely 22 strains were affiliated to known species while the remaining represented 26 new species, *Halopseudomonas* sp. #1 to 5, *Stutzerimonas* sp. #1 to 7 and *Pseudomonas* sp. #1 to 14 (*Pseudomonas* #3 corresponding to *P. chaetocerotis* sp. nov.; Table 1). These results highlight the great genetic diversity, yet unexplored, of strains pertaining to the *Pseudomonadaceae* family within marine samples.

**Table 1:** Phylogenetic affiliation based on ANIb values for the 77 strains, previously not assigned or incorrectly assigned at the species level (Supplementary Table S4). Accession numbers are shown in Supplementary Table S3.

Genus	Group/Subgroup	Species	Strain	Closest type strain	ANIb %	Re-identified species
***Halopseudomonas***						
	*-*	*Pseudomonas* sp.	gcc21	*H. profundi*	87.23	*Halopseudomonas* sp. #1
			5Ae-yellow	*H. neustonica*	98.57	*H. neustonica*
			UBA9879 UBA9027	*H. aestusnigri*	96.92 96.87	*H. aestusnigri*
			EAC28 NAT64 UBA11994 UBA11661	*H. pachastrellae*	95.98* 95.69* 95.66* 95.46*	*Halopseudomonas* sp. #2
			NORP239	*H. salina*	79.38	*Halopseudomonas* sp. #3
			NOPR330	*H. salina*	79.89	*Halopseudomonas* sp. #4
			Lab_640_Crude_bin.6 640_Coassembly_bin.49	*H. laoshanensis*	95.79* 95.71*	*Halopseudomonas* sp. #5
***Stutzerimonas***						
	*-*	*Pseudomonas* sp.	SP133 UBA8086	*S. balearica*	98.26 98.24	*S. balearica*
			MED-G74 MCMED-45	*S. stutzeri*	96.91 96.89	*S. stutzeri*
			MCMED-44 UBA9883 IN1	*S. stutzeri*	89.49 89.30 89.10	*Stutzerimonas* sp. #1
			MT4 MTM4	*S. nitrititolerans*	81.79 81.78	*Stutzerimonas* sp. #2
			SST3	*S. xanthomarina*	82.35	*Stutzerimonas* sp. #3
			Q2-TVGA-2	*S. xanthomarina*	82.29	*Stutzerimonas* sp. #4
			9AG SAT88 UBA9687 UBA9370 UBA9381 SP291 UBA10494	*S. xanthomarina*	87.77 87.80 87.95 87.56 87.82 87.37 87.16	*Stutzerimonas* sp. #5
			MT-1 10B238 UBA9693 IN2	*S. xanthomarina*	87.48 87.47 87.45 87.10	*Stutzerimonas* sp. #6
			IN922 UBA10231 ARS2 SRR3646363	*S. chloritidismutans*	96.32* 96.27* 96.27* 97.07	*S. chloritidismutans*
			NP3	*S. chloritidismutans*	92.54	*Stutzerimonas* sp. #7
***Pseudomonas***						
	*P. oryzihabitans*	*Pseudomonas* sp.	MS15a(2019)	*P. psychrotolerans*	92.81	*Pseudomonas* sp. #1
	*P. aeruginosa*	*Pseudomonas* sp.	AF1 AFW1	*P. aeruginosa*	99.28 99.28	*P. aeruginosa*
	*P. alcaligenes*	*Pseudomonas* sp.	8AS	*P. campi*	88.34	*Pseudomonas* sp. #2
	*P. oleovorans*	*Pseudomonas* sp.	**536**	***P. chengduensis***	**92.03**	***Pseudomonas* sp. #3**
		*Pseudomonas* sp.	8O	*P. chengduensis*	92.39	*Pseudomonas* sp. #4
		*Pseudomonas* sp.	ARS733	*P. chengduensis*	93.91	*Pseudomonas* sp. #5
		*Pseudomonas* sp.	MCMED-46	*P. chengduensis*	96.56	*P. chengduensis*
	*P. anguilliseptica*	*Pseudomonas* sp.	MS19 J237 Gammapro4	*P. marincola*	97.32 97.49 97.28	*P. marincola*
		*Pseudomonas* sp.	UBA2684 UBA10810	*P. benzenivorans*	84.29 84.34	*Pseudomonas* sp. #6
		*Pseudomonas* sp.	8BK	*P. anguilliseptica*	95.96*	*Pseudomonas* sp. #7
		*Pseudomonas* sp.	9AZ	*P. peli*	95.77*	*Pseudomonas* sp. #8
	*P. japonica*	*Pseudomonas* sp.	SRR3933266	*P. qingdaonensis*	98.91	*P. qingdaonensis*
	*P. reidholzensis*	*P. putida*	KT-27	*P. shirazensis*	99.00	*P. shirazensis*
	*P. vlassakiae*	*P. putida*	KH-21-134 KH-21-114	*P. vlassakiae*	91.64 91.63	*Pseudomonas* sp. #9
	*P. putida*	*P. putida*	KT-90 KH-20-11 KH-18-2 IOFA1	*P. alloputida*	94.98 94.96 94.98 95.08	*Pseudomonas* sp. #10
	*P. fragi*	*Pseudomonas* sp.	TAA207 TAD18	*P. weihenstephanensis*	97.11 97.12	*P. weihenstephanensis*
		*Pseudomonas* sp.	9.1(2019)	*P. paraversuta*	99.26	*P. paraversuta*
	*P. koreensis*	*Pseudomonas* sp.	MD195_PC81_125	*P. koreensis*	91.06	*Pseudomonas* sp. #11
		*Pseudomonas* sp.	ef1	*P. siliginis*	96.12*	*Pseudomonas* sp. #12
	*P. gessardii*	*P. fluorescens*	PF08	*P. shahriarae*	99.29	*P. shahriarae*
		*Pseudomonas* sp.	SXM-1	*P. yamanorum*	93.81	*Pseudomonas* sp. #13
		*Pseudomonas* sp.	TAE6080	*P. brennerii*	97.07	*P. brennerii*
	*P. fluorescens*	*P. fluorescens*	B98SK52 B98SM8 B98C39	*P. paracarnis*	99.00 98.94 98.99	*P. paracarnis*
		*Pseudomonas* sp.	BTN1		98.46	
		*Pseudomonas* sp.	J380	*P. carnis*	98.45	*P. carnis*
		*Pseudomonas* sp.	B29B	*P. aylmerense*	98.48	*P. aylmerense*
		*Pseudomonas* sp.	NORP76	*P. veronii*	88.87	*Pseudomonas* sp. #14

*dDDH >70%.

### *Pseudomonas chaetocerotis* sp. nov.

The highest ANIb values between strain 536 and the type strains from the *P. oleovorans* group were observed when compared to *P. chengduensis* DSM 26382^T^ (92.04%) and *P. toyotomiensis* JCM 15604^T^ (91.92%; Supplementary Table S4). dDDH calculations confirmed the new species assignment with values below 70% for hybridization with *P. chengduensis* DSM 26382^T^ (47.40%) and *P. toyotomiensis* JCM 15604^T^ (47.20%). *P. chengduensis* DSM 26382^T^ and *P. toyotomiensis* JCM 15604^T^ were thus selected as reference strains for biochemical and chemotaxonomic characterization. The genomic features of strain 536 are: genome size 5.3 Mbp, scaffold count 44 and gene count 5,113 (coding 4,980; NCBI Prokaryotic Genome Annotation Pipeline, PGAP). Cells of strain 536 were 2.02–3.07 μm long and 0.82–1.19 μm wide, facultative anaerobic, Gram negative, and motile with a single polar flagellum (Supplementary Figure S2). *Pseudomonas* strain 536 was able to grow at 16–41°C, at pH 5–10 and in the presence of 0–8% (w/v) NaCl. Colonies appeared are small green colonies on TCBS and small cream colonies on MA after 48 h at 25°C; and as smalgram.

l, irregular, pale irregular colonies on LB agar after 24 h at 30°C. The biochemical characteristics of strain 536^T^ and the two references strains *P. toyotomiensis* JCM 15604^T^ and *P. chengduensis* DSM 26382^T^ are detailed in Supplementary Table S5. Strain 536^T^ can be easily differentiated from the two nearest relatives by a negative oxidase test or by growth on medium with antibiotic Aztreonam. The fatty acid profile of strain 536^T^ is shown in Supplementary Table S6. The major fatty acid are C18:1 ω7c (35.81%), C16:1 ω7c/*iso*-C15:0 2-OH (22.90%), C16:0 (15.69%) and C12:0 (8.68%), a pattern similar to the two closest type strains, *P. toyotomiensis* JCM 15604^T^ and *P. chengduensis* DSM 26382^T^. The respiratory quinones of strain 536^T^ are Q-9 (97%), Q-8 (2%) and Q-10 (1%). The *rpoD* and whole genome analyses confirmed these affiliations and phenotypic characteristics further discriminated strain 536^T^ from their closest phylogenetic neighbors *P. toyotomiensis* JCM 15604^T^ and *P. chengduensis* DSM 26382^T^. Finally, chemotaxonomic phenotypic and genomic characteristics allowed the distinction from previously described species in the *P. oleovorans* group and thus, the description a new species, *Pseudomonas chaetocerotis* sp., with *Pseudomonas* strain 536^T^ (=LMG 31766^T^ = DSM 111343^T^) as the type strain (protologue; Table 2; Appendix A).

**Table 2:** Protologue of *Pseudomonas chaetocerotis* 536^T^.

Genus name	*Pseudomonas*
Species name	*Pseudomonas chaetocerotis*
Specific epithet	*chaetocerotis*
Species status	sp. nov.
Species etymology	*Pseudomonas chaetocerotis* (chae.to.ce.ro’tis. N.L. gen. n. *chaetocerotis* of the diatom genus *Chaetoceros*).
Description of the new taxon and diagnostic traits	Cell are Gram-stain-negative, motile, rod-shaped, 2.02–3.07 μm long and 0.82–1.19 μm wide. Colonies on LB agar are yellow, flat and irregular. Able to grow at pH 5–10, at 16–41°C and in the presence of 0–8% of NaCl. Positive for nitrate reduction, assimilation of glucose, mannitol, potassium gluconate, capric acid, malate and trisodium citrate, but oxidase and urease negative. In the Biolog GN system (GEN III), positive for utilization of Tween 40, glycyl-L-proline, D-galacturonic acid, methylpyruvate, ɣ-aminobutyric acid, L- alanine, L-galactonic acid lactone, D-gluconic acid, L-lactic acid, β-hydroxy-D, L-butyric acid, L-aspartic acid, D-glucuronic acid, citric acid, L-glutamic acid, α-ketoglutaric acid, L-histidine, mucic acid, propionic acid, L-pyroglutamic acid, quinic acid, L-malic acid, acetic acid, L-serine, D-saccharic acid. Negative for utilization of the following carbon sources: raffinose, sorbitol, lactose, mannose, maltose, melibiose, arabitol, trehalose, myo-inositol, cellobiose, salicin, 3-methylglucose, glycerol, D-glucose-6-phosphate, sucrose, turanose, L-rhamnose, stachyose, *p*-hydroxyphenylacetic acid, D-lactic acid methylester, β-methyl-D-glucoside, α-ketobutyric acid, gentiobiose, *N*-acyl-D-glucosamine, *N*-acetyl-β-D-mannosamine, N-acetyl-D-galactosamine, D-aspartic acid, N-acetyl neuraminic acid and inosine. Weak reactions were observed for the utilization of dextrin, mannitol, fructose, α-hydroxybutyric acid, galactose, L-arginine, L- and D-fucose, glucuronamide, acetoacetic acid, D-fructose-6-phosphate, D-malic acid, D-serine, bromosuccinic acid and formic acid. The predominant fatty acids are C16:0, C18:1 ω7*c* and C16:1 ω7*c*/*iso*-C15:0 2-OH. The predominant ubiquinone is Q-9.
Country of origin	France
Region of origin	Occitanie
Other	Non-axenic culture of *Chaetoceros calcitrans*
Sampling date	17-05-2018
16S rRNA gene accession number	MW333026
Genome accession number	JACFYX000000000
Genome status	Complete (Draft)
Genome size	5.3
GC mol%	62.4
Number of strains in study	2
Source of isolation of non-type strains	Non-axenic culture of *Chaetoceros gracilis*
Information related to the Nagoya protocol	Not applicable
Designation of the type strain	536^T^
Strain collection numbers	LMG 31766^T^ = DSM 111343^T^

### Proposal of transfer of species to the novel genera

We propose the transfer of 3 species, not transferred by Rudra and Gupta, 2021, to the genus *Halopseudomonas* that belong to the *P. pertucinogena* group in our study and in the corresponding references [*P. laoshanensis* (Wang et al., 2021), *P. nanhaiensis* (Pang et al., 2021) and *P. yangmingensis* (Wong and Lee, 2014)]. These transfers are also supported by results of Girard et al. (2020a,b), Girard et al. (2021), and Appendix A.

### Genomics of secondary metabolites production by marine *Pseudomonadaceae*

**Figure 2:**
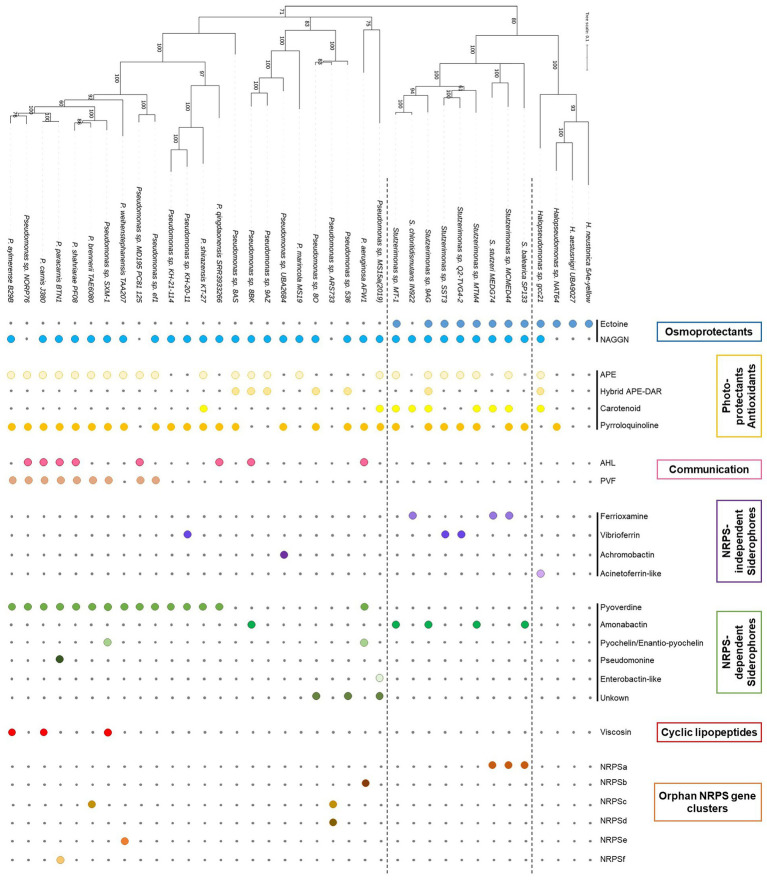
Genes and clusters involved in secondary metabolites production. The presence of a gene/cluster is symbolized by a colored circle, while the absence is indicated by a grey dot. NRPS, Non-ribosomal peptide synthetase.

A sub-selection of 37 genomes, excluding identical strains (i.e., ANIb values >99%), were surveyed for BGCs involved in secondary metabolite production. BGCs found in these 37 genomes included osmoprotectants, photo-protectants, quorum sensing molecules, siderophores, cyclic lipopeptides (CLPs) and numerous orphan NRPS gene clusters. Results are presented in Figure 2.

#### Osmoprotectants

Osmoprotection seems to be a fundamental mechanism to cope with high salinity level exhibited in marine ecosystems. Early on, N-acetylglutaminylglutamine amide (NAGGN) was shown to be a dominant osmolyte used by *Pseudomonas* spp. when subjected to osmotic stress (D’Souza-Ault et al., 1993; Pocard et al., 1994). Lately, the NAGGN cluster was show to be widespread in *Pseudomonas* (Alam et al., 2021) and, more generally, in bacterial genomes (Sagot et al., 2010). Interestingly, while the NAGGN cluster is present in the genome of almost all *Pseudomonadaceae* strains, it seems that the ectoine BGC is confined to *Halopseudomonas* and *Stutzerimonas* strains (Figure 2). The original ectoine BGC, *ectABC*, encodes for a diaminobutyric acid (DABA) acetyltransferase (EctA), DABA aminotransferase (EctB) and ectoine synthase (EctC). However, numerous operon variants have been identified in halophilic γ-Proteobacteria, including an *ectR* (MarR-type transcriptional regulator gene), an *ectD* (ectoine hydroxylase gene) and/or an *ask* (aspartate kinase) (Schwibbert et al., 2011). *Stutzerimonas stutzeri* (*P. stutzeri*) was shown to produce hydroxyectoine *via* a *ectABCD-ask* cluster as observed in all *Stutzerimonas* genomes (Supplementary Table S7; Seip et al., 2011). On the other hand, the ectoine BGC present in *Halopseudomonas* strains were either an *ectABCD-ask*, like *Stutzerimonas* strains, or an *ectABC-ask* similarly to *Vibrio parahaemolyticus* (Supplementary Table S7), indicating the possible production of the osmolyte hydroxyectoine or ectoine. A phylogeny of the *ectBC* genes showed high similarity with the *rpoD* phylogeny revealing that this BGC has evolved in accordance to the evolutionary history of both genera (Supplementary Figure S3). However, two *Stutzerimonas* strains, *S. nosocomialis* and *S. kirkiae*, were accommodating a shorter version of this BGC, respectively, *ectBCD-ask* and *ectBC-ask* and were standing out of the *ectBC* phylogeny, indicating a recent acquisition/modification of this cluster (V3/V4; Supplementary Figure S3).

#### Photoprotectants/antioxidant

Antioxidants have a great application potential particularly in health and food industry and bacteria were recently pinpointed as a cost-effective way to produce this type of compounds (Ram et al., 2020). Antioxydants can also turn to be potential antimicrobials or quorum sensing inhibitors (Gökalsın et al., 2017; Ram et al., 2020). Among them, carotenoids have potential application in cancer prevention, reversal of multidrug resistance, reduction of virulence (*via* quorum quenching) but also as additives in food industry (Ram et al., 2020). Carotenoid production from *Pseudomonas* strains has been reported (Beuttler et al., 2011) and novel chemical structure were discovered using marine *Pseudomonas* (i.e., sponge associated isolate; Okadaxanthin; Miki et al., 1994). We observed in our genomes two type of Zeaxanthin-like BGCs, the *crtE-idi-XYIBZ* organization, found both in *Pseudomonas*, *Halopseudomonas* and *Stutzerimonas* strains, as previously reported for *Enterobacteriaceae* strains (Sedkova et al., 2005), and the unique organization *crtE-idi-XYIB*, found in *P. shirazensis* KT-27 (Supplementary Table S8). In other strains either the *crtE* upstream, the *idi* or the *crtIBZ* downstream the *crtY* are incomplete or missing, probably due to the fact that some genomes are highly fragmented.

**Figure 3:**
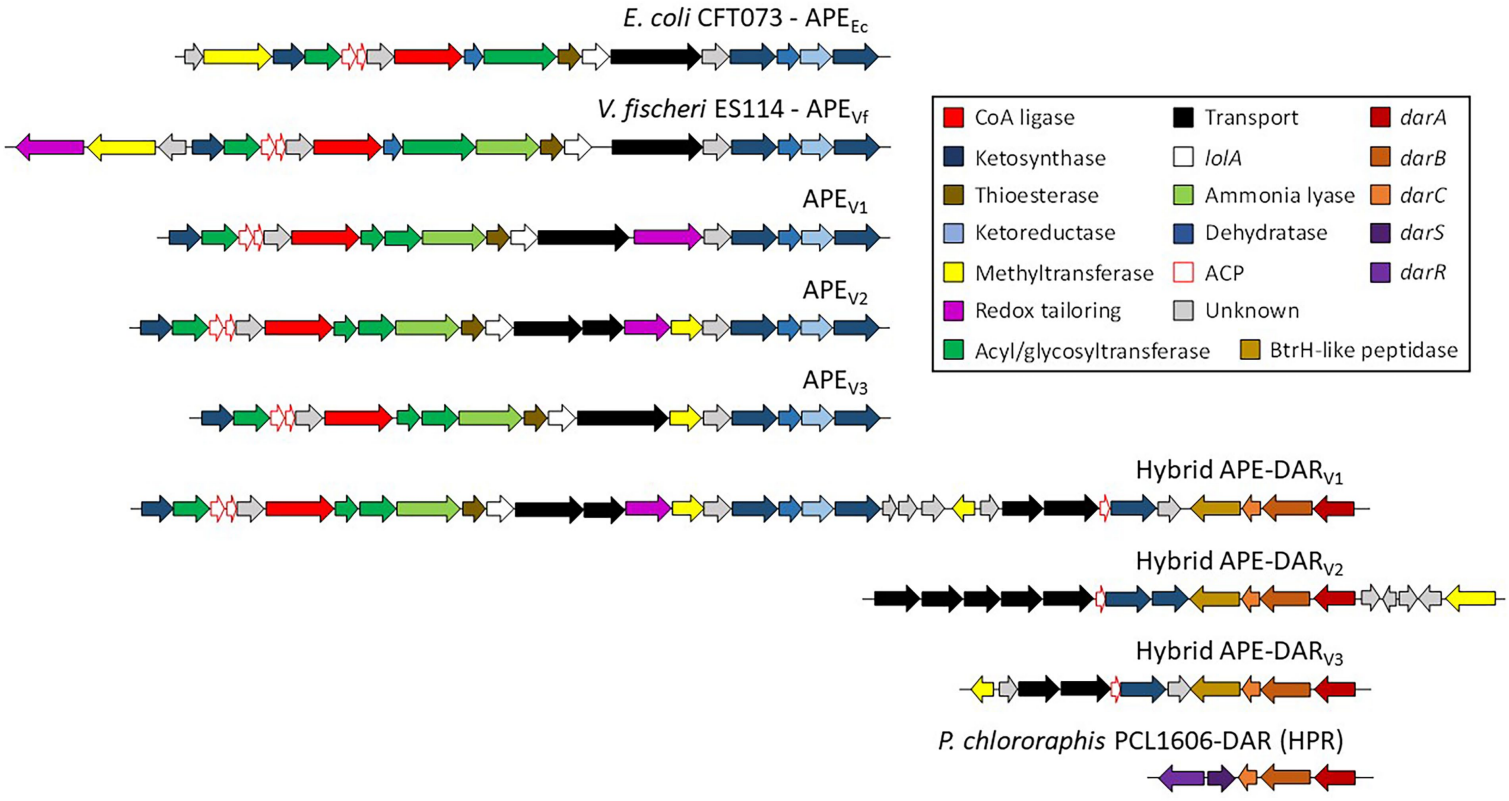
Known and proposed gene clusters involved in the biosynthesis of APE, DAR and Hybrid APE-DAR. All genes are scaled to the depicted size.

Aryl polyenes (APE) is a highly abundant class of natural products that are functionally related to anti-oxidative carotenoids and APE BGCs are widespread among bacteria (Cimermancic et al., 2014; Schöner et al., 2016). So far, four classes of APEs have been described, the xanthomonadin and derivatives, the hybrid APE – dialkylresorcinol (DAR) arcuflavin, the hybrid APE-DAR flexirubin and derivatives and a flexirubin-like APE found in *E. coli* and *V. fischeri* (Cimermancic et al., 2014; Schöner et al., 2016). We found different variants of the APE BGCs in most of the analyzed genomes, all pertaining to the fourth class with organizations similar to *E. coli* and *V. fischeri* (APE_V1-V3_; Figure 3). Interestingly, we also found three types of hybrid APE-DAR BGCs with unique organizations and compositions that cannot be affiliated to the known classes (hybrid APE-DAR_V1-V3_; Figure 3; Cimermancic et al., 2014; Schöner et al., 2014). Particularly, the type strain of *P. chaetocerotis* sp. nov., as well as *Pseudomonas* sp. 8O, carry a unique hybrid APE-DAR BGC, with an APE BGC similar to *E. coli* and *V. fischeri* connected to a DAR BGC (APE-DAR_V1_; Figure 3). Likewise, the DAR part of these hybrid APE-DAR BGCs diverge from the 2,5 dialkylresorcinol (HPR) BGC, well-known for its antifungal activity, a biocontrol asset for crop associated *Pseudomonas* (HPR, Figure 3; Calderón et al., 2014; Biessy et al., 2019). Thus, a conscientious work of chemical characterization needs to be done to investigate the structural diversity of this type of compounds. Furthermore, even if it was recently demonstrated that APE genes are essential for *E. coli* to form biofilms (Johnston et al., 2021), little is known about the biological and ecological functions of this metabolite family.

Pyrroloquinoline quinone (PQQ) was shown to be a plant growth promoting factor produced by *P. fluorescens*. The *pqqFABCDEMKJIH* cluster was identified as the source of this metabolite and it was demonstrated that all genes were essential for the production of PQQ (Choi et al., 2008). A truncated version of this cluster, *pqqFABCDEMIH*, is also present in *P. putida* KT2440 and is involved in the production of gluconic acid allowing the solubilization of mineral phosphates (An and Moe, 2016). PQQ BGCs are widespread in *Pseudomonadaceae* genomes and the classical organization *pqqABCDE* was conserved among the analyzed genomes, however many variants (i.e., presence or absence of the “accessory” genes *pqqFHIJKM*) were identified and potentially represent new forms of PQQ with similar or unknown functions (Supplementary Table S8).

#### Cell-to-cell communication

Quorum sensing (QS) genes were only found in *Pseudomonas* and were absent from *Halopseudomonas* and *Stutzerimonas* genomes. All LuxI homologs identified in this study are clustering with known *Pseudomonas* AHL synthases (Supplementary Table S8; Supplementary Figure S4). One divergent LuxI homolog was identified in *Pseudomonas* sp. 8BK and, interestingly, this is the first study reporting QS in strains pertaining to the *P. anguilliseptica* group (Supplementary Figure S4). Only two strains harbored two QS gene pairs, *P. aeruginosa* AFW1 carried the classical *lasI/R* and *rhlI/R* gene pairs, likewise most *P. aeruginosa* strains (Juhas et al., 2005), and *Pseudomonas* sp. NORP76, pertaining to the *P. fluorescens* subgroup, with one LuxI clustering with PmrI from *P. wayambapalatensis* RW10S2 and the second with PfsI from *P. fuscovaginae* UBP0736. Multiple communication systems, homologous to the *luxI/luxR* gene pair, have been identified among *Pseudomonas* genomes (Supplementary Table S9; Juhas et al., 2005). *N*-acyl-homoserine lactones (AHLs) based communication regulates numerous biological functions (e.g., virulence, biofilm formation) in *Pseudomonas* spp., but also the production of secondary metabolites (e.g., mupirocin, phenazine, cyclic lipopeptides) (Chin-A-Woeng et al., 1998; El-Sayed et al., 2001; Dubern et al., 2006; Arp et al., 2018). In marine environments, AHL were also shown to mediate interactions with cyanobacteria (Van Mooy et al., 2012), thus further studies are needed to chemically characterize AHL diversity among marine *Pseudomonas.*

Pyrazine-derived compounds are well known to coordinate communal behavior among bacteria (Silva-Junior, 2018). *Pseudomonas* strains typically produce pyrazine N-oxides (PNOs) through the *Pseudomonas* virulence factor BGC (pvfABCD) and PVF autoinducers regulate the expression of many genes involved in virulence, colonization and competition (Kretsch et al., 2018, 2021). Pvf genes are widely distributed among *Proteobacteria* and have been identified, in this study, among strains belonging to the *P. fluorescens* group (Figure 2; Supplementary Table S10). In marine environments, the autoinducer 3,5-dimethylpyrazin-2-ol (DPO) regulate virulence factor production and biofilm formation (Eickhoff and Bassler, 2018). Recently, DPO was also described to mediate interactions with bacteriophages (Silpe and Bassler, 2019; Silpe et al., 2020). Together both AHLs and PVF autoinducers may be the key to understand the interactions between *Pseudomonas* and other organisms in marine environments.

#### Siderophores

**Figure 4:**
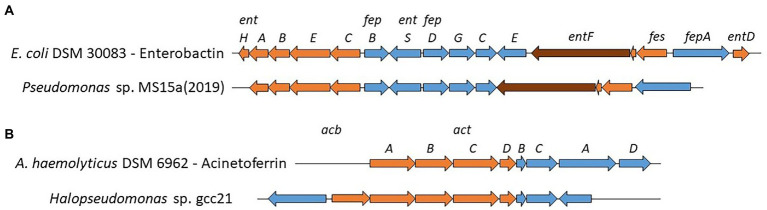
Known and proposed gene clusters involved in the biosynthesis of Enterobactin **(A)** and Acinetoferrin **(B)**. All genes are scaled to the depicted size. Biosynthesis genes are highlighted in orange (NRPS in brown) and transport genes in blue.

Oceans are an iron limiting environment and siderophore production is a major mechanism for marine heterotrophic bacteria but it also plays an important role in the oceanic biogeochemical cycling of iron (Mawji et al., 2008). Numerous NRPS-dependent and independent BGCs for siderophore production were identified in our marine *Pseudomonadaceae* genomes. We observed a greater diversity of siderophores BGCs among *Pseudomonas* strains (*n* = 8). Several BGCs are very common in *Pseudomonas* genomes, such as pyoverdines, pyochelin, enantio-pyochelin or pseudomonine (Gross and Loper, 2009; Garrido-Sanz et al., 2016), while others have never been detected or sporadically in very few *Pseudomonas* strains. Indeed, vibrioferrin production was previously reported in *P. fragi* (Stanborough et al., 2018) and achromobactin was characterized in *P. syringae* strains (Berti and Thomas, 2009; Owen and Ackerley, 2011) while amonabactin has never reported in *Pseudomonas* strains. Interestingly, *Pseudomonas* sp. MS15a(2019), carries an enterobactin-like cluster, excluding, in comparison to the original cluster present in *E.coli* DSM 30083, the presence of three genes (*fepE*, *entD* and *entH*, Figure 4A). Enterobactin production has never been reported for *Pseudomonas* strains, however the ClusterBlast function of antismash allowed us to identify identical (*P. oryzihabitans* S00005, *P. oleovorans* AG1002, *Pseudomonas* sp. Snoq117.2 and 1766) or similar clusters in many *Pseudomonas* genomes.

The most common NRPS-based siderophores among fluorescent *Pseudomonas* are the pyoverdines (Meyer et al., 2008; Biessy et al., 2019). A first attempt of classification was made based on pyoverdines secreted by *P. aeruginosa* strains and three classes were defined. Later on, the structural characterization of a large amount of pyoverdines have led to the expansion of these classes (Cezard et al., 2014). The structural diversity of pyoverdines is well characterized, and they all consist of three distinct structural parts, a quinoline-1-carboxylic acid containing a chromophore, a dicarboxylic acid or its monoamide, and a peptide chain comprising 6–14 amino acids (Visca et al., 2007). Both the chromophore and the peptide chain of pyoverdines are synthesized by NRPSs. While the chromophore part, encoded by *pvdL*, is identical and common to all *Pseudomonas* strains, the primary difference among pyoverdines lies in their peptide chain and thus the NRPSs organizations. Initial studies on *P. aeruginosa* identified 3 NRPS genes, namely *pvdIJD*, encoding for 8 modules (4, 2 and 2) (Ravel and Cornelis, 2003). Subsequently, diverse pyoverdine BGCs were described in *Pseudomonas* strains (e.g., *P. syringae*, *P. putida* and *P. fluorescens*) differing in the number of gene, gene organization, modules composition and thus peptide chain length and composition (Ravel and Cornelis, 2003; Ye et al., 2013). Each module contains domains with different functions, with the essential ones being the condensation domain (C-domain, formation of the peptide bond), thiolation (T-domain) and peptide carrier protein (PCP), and adenylation domain (A-domain, amino acid selection) (Ye et al., 2013). A-domains, allowing with a certain specificity the selection and sequential incorporation of amino acids into the peptidic chain, enable to predict the sequence of the peptide (Bachmann and Ravel, 2009). All strains pertaining to the *P. fluorescen*s and *P. putida* groups and the *P. aeruginosa* AFW1 have a pyoverdine BGC (Supplementary Table S10). A-domains phylogeny, including *Pseudomonas* strains with characterized pyoverdines (Supplementary Table S11) and A-domains extracted from the genomes of our marine strains (Supplementary Table S10), allowed the prediction of amino-acid peptide sequence for 9/15 strains (Table 3; Supplementary Figure S5). The remaining strains have a fragmented pyoverdine BGC making the predictions impossible. Most of the predicted amino-acid peptide sequence were identical to previously described type I and II pyoverdines. Nonetheless, two unusual peptide sequence were identified in *Pseudomonas* sp. KH-21-114 (Lys-Asp-Ser-Orn) and *P. shirazensis* KT-27 (Ser-Lys-His-Asp-Orn), and such short peptide sequence have never been described. However, further studies are needed to determine if these pyoverdines are actually produced and remain functional. Another unusual BGC coding for a NRPS-based siderophore was identified in the genome of three strains, *Pseudomonas* sp. MS15a(2019) (*P. oryzihabitans* group), *Pseudomonas* sp. 8O and *P. chaetocerotis* 536^T^ (*P. oleovorans* group). A-domains phylogeny allowed the prediction of a peptide composed of 6 amino acids Asp-Dab-Ser-Orn-Ser-Orn (not done for *P. chaetocerotis* 536^T^, second part of the BGC too fragmented). An identical BGC was shown to be responsible for the production of an hydroxamate-based siderophore by *P. mendocina* ymp, however this siderophore still awaits chemical characterization (Awaya and DuBois, 2008).

**Table 3:** Siderophores peptide amino-acid sequences predictions.

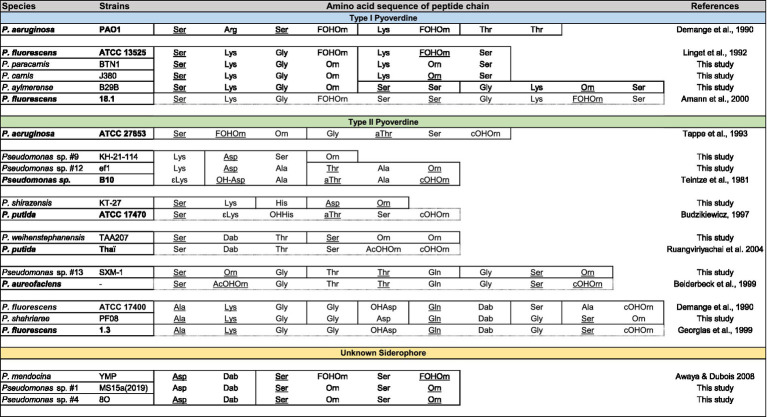

Strains for which the amino-acid sequence was previously confirmed by chemical characterization are highlighted in bold. Rectangles are representing gene organization and dotted rectangles indicate strains for which gene orgnization is unkown. D-amino-acids are underlined.

On the other hand, less diversity was observed in *Stutzerimonas* strains (*n* = 3) while only one strain of *Halopseudomonas* carried a siderophore BGC. *Stutzerimonas* strains carried, as previously reported, either an amonabactin, a ferrioxamine or a vibrioferrin BGC (Zawadzka et al., 2006; Essén et al., 2007). *Halopseudomonas* sp. gcc21, possess an acinetoferrin-like BGC, with the *actA* gene upstream the biosynthesis genes (orange Figure 4B) and an extra biosynthesis gene upstream *acbA* coding for a pyridoxal-dependent decarboxylase (Pfam: PF0082). Interestingly, there are no hits (ClusterBlast, antismash) with other *Halopseudomonas* strains but with *Alkanindiges illinoisensis* DSM 15370 and *Acinetobacter* strains. Considering the fact that no other *Halopseudomonas* strain carries such siderophore BGC, we believe that this acinetoferrin-like BGC has been acquired recently in the evolution of this strain.

#### Cyclic lipopeptides

CLPs are biosurfactants made of a fatty acid tail attached to a cyclic oligopeptide with a wide range of antibacterial and antifungal activities (Geudens and Martins, 2018; Götze and Stallforth, 2019). *Pseudomonas* CLPs are involved numerous ecological functions such as biocontrol activity, bacterial motility or biofilm formation (Geudens and Martins, 2018). Similarly to pyoverdines, CLPs are assembled by NRPSs and from the modularity of these enzymes comes a wide diversity of variants, classified in several families based on the size and nature of their oligopeptide (Geudens and Martins, 2018). CLP BGCs are absent from *Halopseudomonas* and *Stutzerimonas* genomes. Most *Pseudomonas* strains analyzed here do not carry CLP BGCs but their presence was revealed in the genomes of three strains from the *P. fluorescens* group, namely *Pseudomonas* sp. SXM-1, *P. carnis* J380 and *P. aylmerense* B29B. All BGCs were composed of three NRPS genes, coding, respectively, for 2, 4 and 3 modules, in a split organization where the first biosynthetic gene is separated from the two others (Supplementary Table S10). This allowed us to conclude their affiliation to the Viscosin family and a phylogenetic analysis based on the concatenated NRPS proteins, including known members of the Viscosin family (Supplementary Table S12) and the three strains cited above, is shown in Supplementary Figure S6. Members of this CLP family are well-known to be involved, for soilborne and plant associated *Pseudomonas*, in swarming motility and antagonism, but their function within marine ecosystems remains unknown.

#### Unknown clusters

We detected numerous putative NRPS clusters, one among *Stutzerimonas* strains (NRPSa) and 5 among *Pseudomonas* strains (NRPSb to f, Figure 2). Accession numbers for these NRPSs genes can be found in Supplementary Table S10. To date, most *Pseudomonas* secondary metabolites are NRPS based (Gross and Loper, 2009) and NRPSs are a promising source for the discovery of novel bioactive natural products (Challis, 2008). Beside the putative NRPS clusters, we observed the presence of many RIPP-like genes within the analyzed genomes (1 to 4 RIPP-genes by genome). Ribosomally synthesized and post-translationally modified peptides (RiPPs) are a diverse group of bioactive compounds (Arnison et al., 2013) and RIPP genes are widespread among prokaryotic genomes (Skinnider et al., 2016). These orphan NRPS and RIPP clusters represent a real challenge for microbiologists and chemists in the discovery of new chemical structure and potent biological activities.

## Conclusion

We studied here the genetic diversity together with the metabolic potential of marine *Pseudomonadaceae*. We showed that marine environments host a wide diversity of *Pseudomonadaceae* and highlight the need to further explore their diversity, distribution, and seasonality in marine environments. The identification of BGCs responsible for secondary metabolites production in *Pseudomonas*, *Stutzerimonas* and *Halopseudomonas* genomes allowed us to identify new producers of known metabolites and new variants of BGCs possibly coding for the production of new metabolites. Numerous strains, including the type strain of the newly described species *P. chaetocerotis*, require further work to chemically characterize these new compounds, particularly the new variants of siderophores (pyoverdines, acinetoferrin and enterobactin) and APEs, but also new metabolites such as the hybrid APE-DARs and the new NRPS-dependent siderophore. Finally, the majority of these different classes of metabolites have well-defined ecological functions for *Pseudomonads* in terrestrial environments but a tremendous amount of work is still needed to understand their role and importance within marine ecosystems.

## Data availability statement

The datasets presented in this study can be found in online repositories. The names of the repository/repositories and accession number(s) can be found in the article/Supplementary material.

## Author contributions

LG: conceptualization, data analysis, investigation, and writing – original draft and editing. CL: methodology, data analysis, and writing – review and editing. VN and RM: writing – review and editing and funding acquisition. JB: resources, formal analysis, writing – review and editing, and funding acquisition. All authors contributed to the article and approved the submitted version.

## Funding

This work was funded by Sorbonne Universités (JB), supported by the EOS grant 30650620 (LG and RM). CL was supported by the Research Foundation – Flanders grant 1S64720N and the Research Council of KU Leuven grant PDMt2/21/038.

## Conflict of interest

The authors declare that the research was conducted in the absence of any commercial or financial relationships that could be construed as a potential conflict of interest.

## Publisher’s note

All claims expressed in this article are solely those of the authors and do not necessarily represent those of their affiliated organizations, or those of the publisher, the editors and the reviewers. Any product that may be evaluated in this article, or claim that may be made by its manufacturer, is not guaranteed or endorsed by the publisher.

## Footnotes

^1^ https://www.ncbi.nlm.nih.gov/genome/browse/#!/prokaryotes/13508/

^2^ https://www.ncbi.nlm.nih.gov/genome/?term=pseudomonas+fluorescens

^3^ https://www.ncbi.nlm.nih.gov/genome/browse/#!/prokaryotes/174/

^4^ https://ggdc.dsmz.de/home.php

^5^ http://nrps.igs.umaryland.edu/

## Appendix A: New species descriptions

### Description of *Pseudomonas chaetocerotis* sp. nov. (protologue Table 2)

(chae.to.ce.ro’tis. N.L. gen. n. *chaetocerotis* of the diatom genus *Chaetoceros*)

The type strain is 536^T^ (LMG 31766^T^ = DSM 111343^T^) and was isolated from a culture of *Chaetoceros calcitrans*, Leucate, France in 2018. Its G + C content is 62.40 mol % (calculated based on its genome sequence). The 16 rRNA gene and whole genome sequence of 536^T^ are publicly available through the accession numbers MW333026 and JACFYX000000000, respectively.

### Description of *Halopseudomonas laoshanensis* comb. nov.

(lao.shan.en’sis. N.L. masc./fem. Adj. *laoshanensis*, of Laoshan mountain, referring to the geographical origin of the type strain)

Basonym: *Pseudomonas laoshanensis.*

The description of this species is as given by Wang et al. (2021) for *Pseudomonas laoshanensis.*

Type strain: Y22^T^ = CGMCC 1.16552^T^ = JCM 32580^T^ = KCTC 62385^T^.

### Description of *Halopseudomonas nanhaiensis* comb. nov.

(nan.hai.en’sis. N.L. masc./fem. Adj. *nanhaiensis*, pertaining to Nanhai, a sea in South China where the sample was isolated)

Basonym: *Pseudomonas nanhaiensis.*

The description of this species is as given by Pang et al. (2021) for *Pseudomonas nanhaiensis.*

Type strain: SCS 2-3^T^ = GDMCC 1.2219^T^ = JCM 34440^T^.

### Description of *Halopseudomonas yangmingensis* comb. nov.

(yang.ming.en’sis. N.L. masc./fem. Adj. *yangmingensis*, pertaining to the Yang-Ming National Park, Taiwan, Republic of China, from where the organism was isolated)

Basonym: *Pseudomonas yangmingensis.*

The description of this species is as given by Wong and Lee (2014) for *Pseudomonas yangmingensis.*

Type strain: CRS1^T^ = DSM 24213^T^.
