# Supplementary material for: Genomic diversity and metabolic potential of marine Pseudomonadaceae
Source: Front Microbiol. 2023 Apr 6;14:1071039. doi: 10.3389/fmicb.2023.1071039 (PMC10165715; doi:10.3389/fmicb.2023.1071039)
Supplement: Supplementary file 1 [file Data_Sheet_1.docx]

**Fig. S1**: Maximum likelihood phylogenetic tree based on partial *rpoD* gene (~ 650 bp) including 23 environmental *Pseudomonas* isolates and related type strains from the *P. oleovorans* group. The tree was constructed using the T93+G model (MEGA-X) with 1,000 bootstraps (only bootstrap values higher than 50% are indicated). The *rpoD* sequence of *P. anguilliseptica* LMG 21629^T^ was included as outgroup. Strains indicated in bold were selected for whole genome sequencing. Genbank accession numbers are reported in Table S1 and S2.


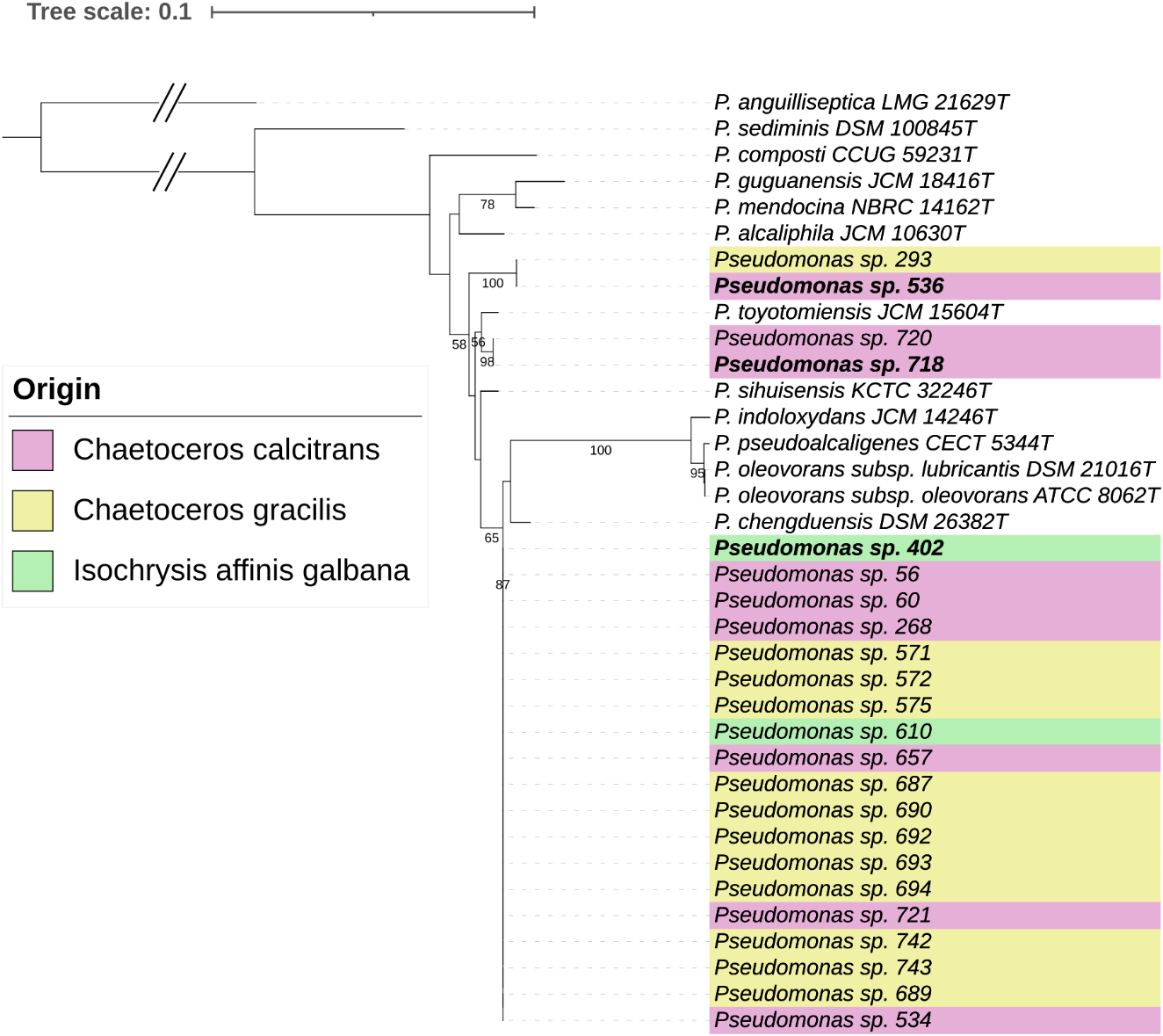


**Fig. S2:** TEM micrograph of negatively stained cells of *Pseudomonas* strain 536^T^ grown in LB for 18h at 30°C, showing the presence of a single polar flagellum.


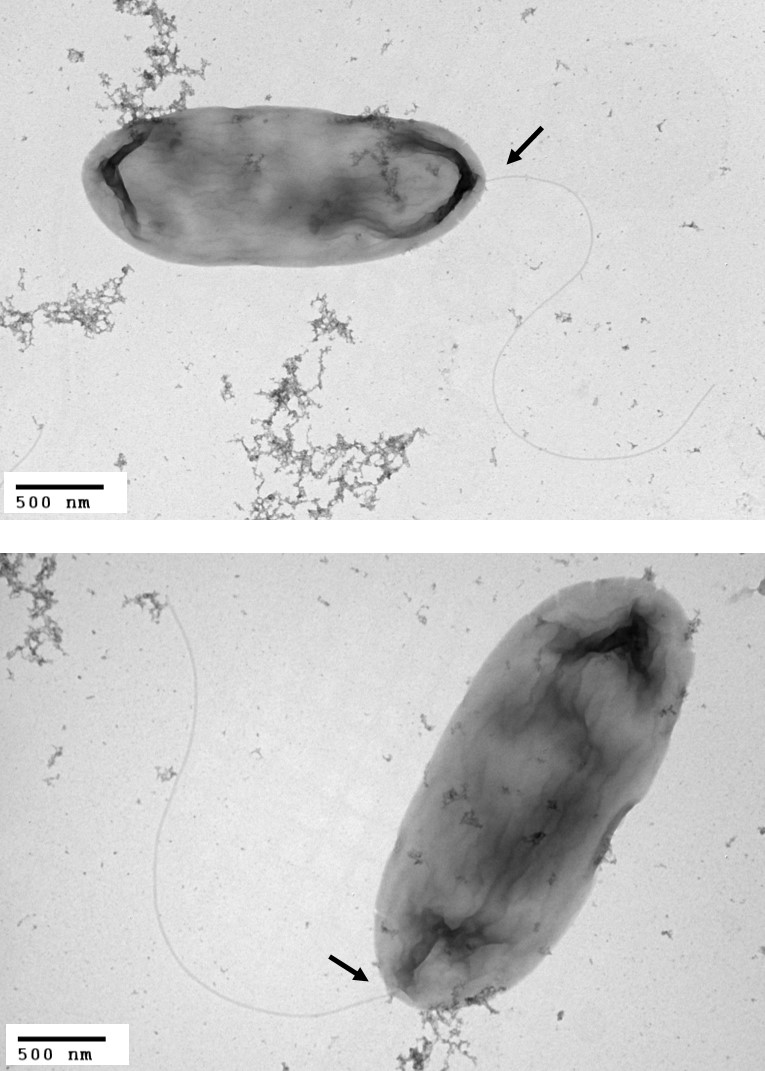

**Fig. S3 :** Phylogenetic tree based on concatenated EctBC proteins (Table S7). Maximum likelihood tree constructed using the LG+I+G model (MEGA-X).
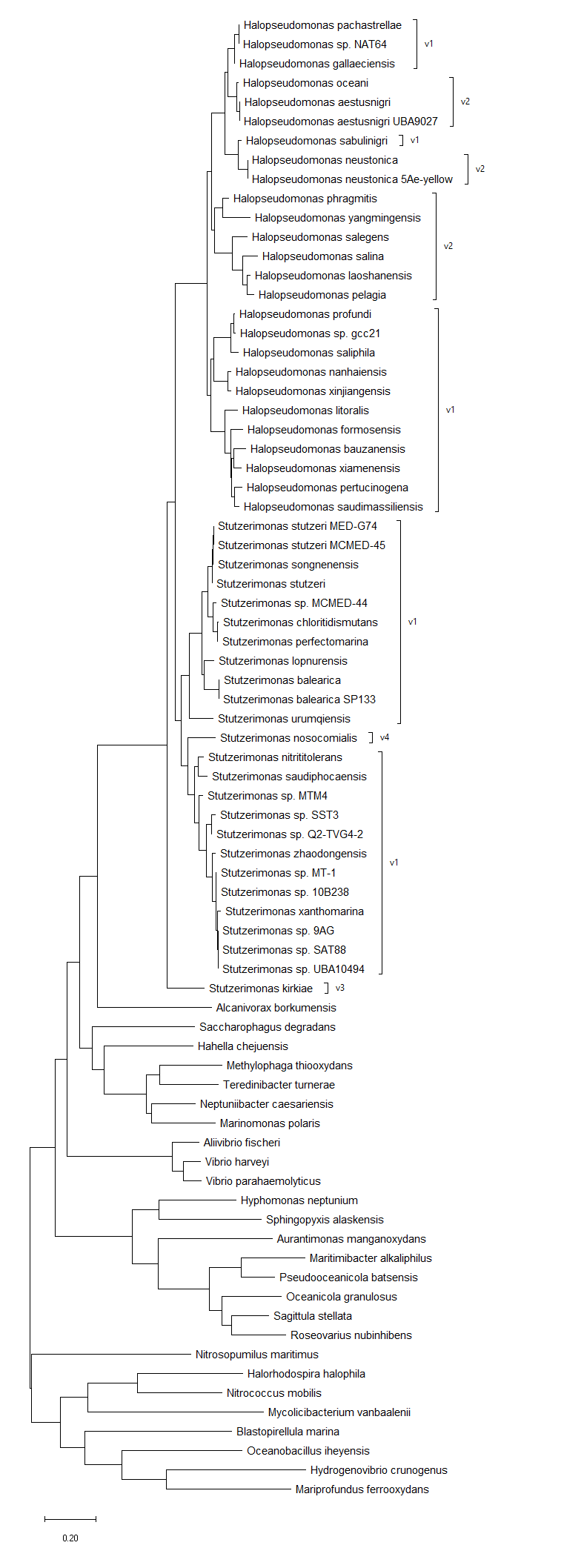


**Fig. S4 :** Phylogenetic tree based on LuxI homologous proteins (Table S8 and S9). Maximum likelihood tree constructed using the JTT+G model (MEGA-X). Environmental strains analyzed in this study are highlighted in bold (Table S8).


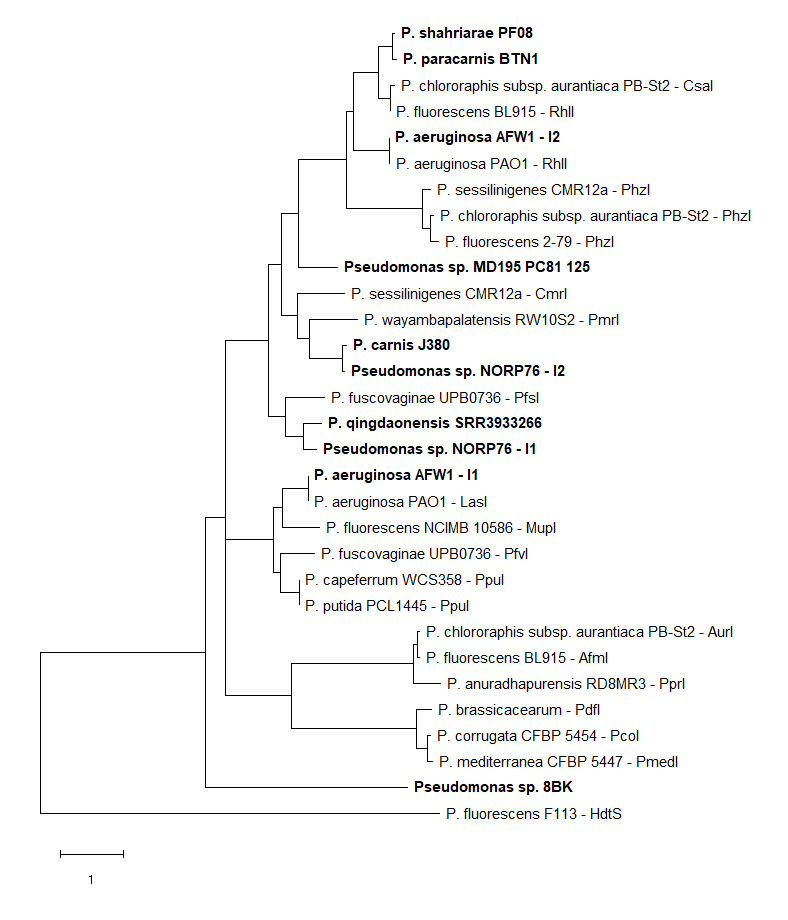


**Fig. S5 :** Phylogenetic tree based on NRPS A-domains used for amino-acid sequence of peptide chain predictions (Table 2). Maximum likelihood tree constructed using the LG+F+I+G model (MEGA-X).


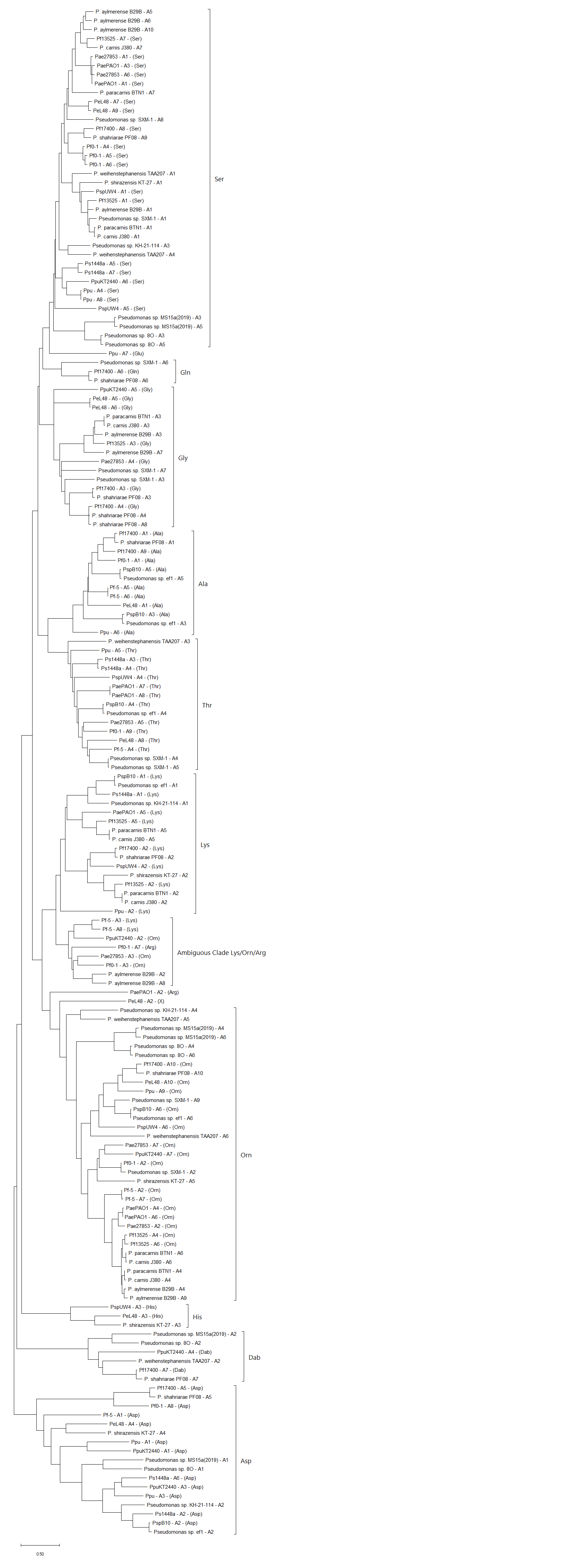


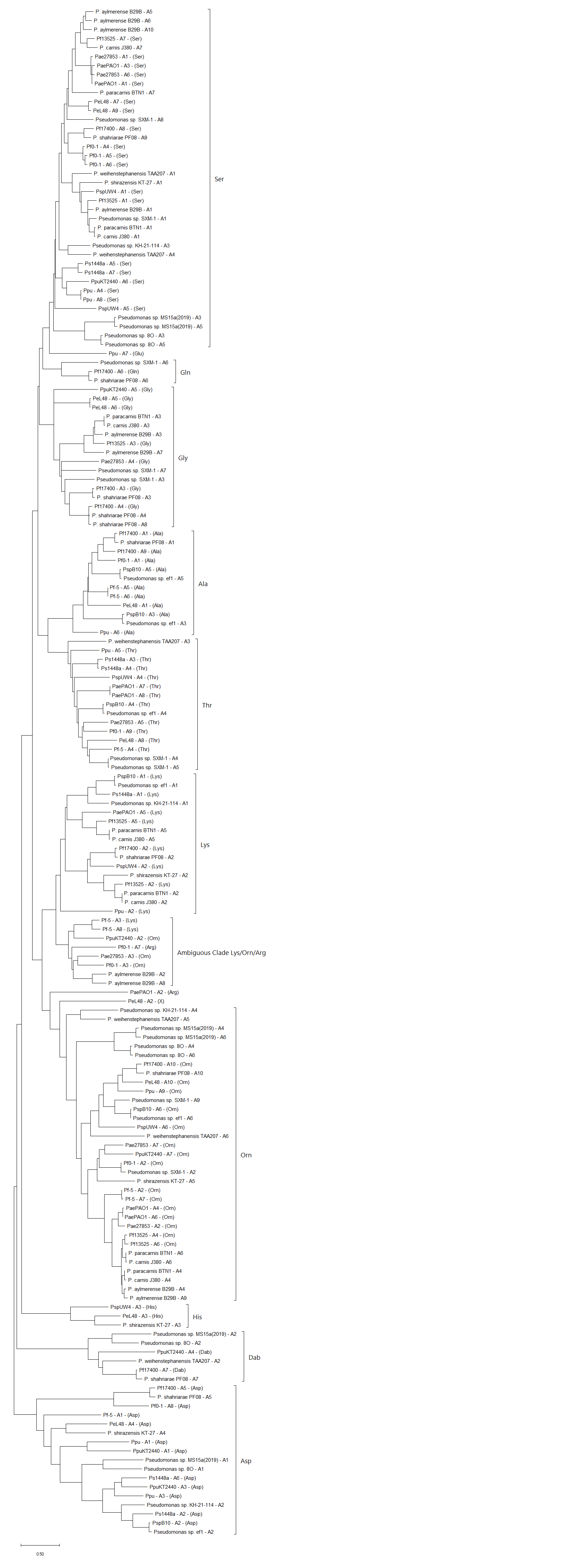


**Fig. S6** Phylogenetic tree based on concatenated NRPS proteins from the Viscosin family. Maximum likelihood tree constructed using the JTT+F+I+G model (MEGA-X). Characterized producers are listed in Table S10.
